# Polymorphism of *XRCC1*, *XRCC3*, and *XPD* Genes and Risk of Chronic Myeloid Leukemia

**DOI:** 10.1155/2014/213790

**Published:** 2014-05-15

**Authors:** Claudia Bănescu, Adrian P. Trifa, Smaranda Demian, Erzsebeth Benedek Lazar, Delia Dima, Carmen Duicu, Minodora Dobreanu

**Affiliations:** ^1^Department of Medical Genetics, University of Medicine and Pharmacy Tirgu Mures, 38 Gh, Marinescu Street, Romania; ^2^Department of Medical Genetics, “Iuliu Hatieganu” University of Medicine and Pharmacy, Cluj-Napoca, Romania; ^3^Hematology Clinic 1, University of Medicine and Pharmacy Tirgu Mures, Romania; ^4^Hematology Clinic 2, University of Medicine and Pharmacy Tirgu Mures, Romania; ^5^Department of Hematology, “Ion Chiricuta” Cancer Institute, Cluj-Napoca, Romania; ^6^Pediatric Clinic, University of Medicine and Pharmacy Tirgu Mures, Romania; ^7^Laboratory Medicine, University of Medicine and Pharmacy Tirgu Mures, Romania

## Abstract

The genetic polymorphisms of X-ray repair cross complementing group 1 (*XRCC1*), X-ray repair cross complementing group 3 (*XRCC3*), and xeroderma pigmentosum complementation group D (*XPD*) repair genes may lead to genetic instability and leukemogenesis. The purpose of the study was to evaluate the association between *XRCC1* Arg399Gln, Arg280His and Arg194Trp, *XRCC3* Thr241Met, and *XPD* Lys751Gln polymorphisms and the risk of developing CML in Romanian patients. A total of 156 patients diagnosed with CML and 180 healthy controls were included in this study. We found no association between CML and *XRCC1* or *XRCC3* variant genotypes in any of the investigated cases. A significant difference was observed in the variant genotype frequencies of the *XPD* Lys751Gln polymorphism between the patients with CML and control group (for variant homozygous genotypes, OR = 2.37; 95% CI = 1.20–4.67; *P* value = 0.016 and for combined heterozygous and variant homozygous genotypes, OR = 1.72; 95% CI = 1.10–2.69; *P* value = 0.019). This was also observed when analyzing the variant 751Gln allele (OR = 1.54; 95% CI = 1.13–2.11; *P* value = 0.008). Our results suggest that the *XPD* Lys751Gln variant genotype increases the risk of CML.

## 1. Introduction

Chronic myeloid leukemia (CML) is a myeloproliferative neoplasm characterised by the Philadelphia chromosome (Ph), a reciprocal chromosomal translocation t (9;22)(q34;q11) leading to the fusion of the Abelson murine leukemia (*ABL*) gene on chromosome 9 with the breakpoint cluster region (*BCR*) gene on chromosome 22 [1].

CML can be classified into distinct clinical phases: chronic phase, accelerated phase, and blast phase. Diagnosis is most commonly established during the chronic phase. The fusion gene* BCR-ABL* in CML results in genomic instability and defective repair that can lead to acquisition of genomic changes [2].

DNA damage repair pathways are important for removing different types of DNA damage. The base excision repair (BER), nucleotide excision repair (NER), and double strand break repair (DSB repair) are the most important DNA repair pathways [3]. Mutations are early events in carcinogenesis and impaired DNA repair might be a risk factor for many cancers [4].

Common genetic polymorphisms in DNA repair genes might affect protein function and thus the capacity of repair DNA damage, which in turn could lead to genetic instability and leukemogenesis. Polymorphisms in DNA repair genes are thought to be a risk factor for cancer as a result of increased rate of mutations.

Among them, polymorphisms of X-ray repair cross complementing group 1 (*XRCC1*), X-ray repair cross complementing group 3 (*XRCC3*), and xeroderma pigmentosum complementation group D (*XPD*) have been studied extensively.

DNA lesions caused by internal and external factors such as ionizing radiation, alkylating agents, and oxidation repaired through the base excision repair pathway (BER). BER is one of the four major DNA repair pathways [3].

The nucleotide excision repair (NER) pathway is responsible for repair of lesions such as bulky adducts and thymidine dimers [5]. Double strand break (DSB) repair is responsible for the repair of double strand DNA breaks produced by exogenous agents (such as ionizing radiation and some chemotherapeutic drugs) and endogenous formed reactive oxygen species. One of the main pathways for the repair of DNA double strand breaks is homologous recombination (HR), which is important in DNA repair occurring during cellular replication [6].

Several single-nucleotide polymorphisms (SNPs) in* XRCC1*,* XRCC3,* and* XPD *genes have been identified. Among them,* XRCC1* Arg399Gln, Arg280His, and Arg194Trp,* XRCC3* Thr241Met, and* XPD* Lys751Gln polymorphisms are the most studied in cancers, including leukemia.

X-ray cross complementing gene 1 (*XRCC1*) is one of the most important genes involved in DNA repair, specifically in the base excision repair pathway and in single-strand break repair activity [7, 8]. The* XRCC1 *gene encodes a protein that is associated with DNA polymerase beta, DNA ligase III, and poly ADP-ribose polymerase (PARP) and functions in a complex to facilitate the repair of the damaged bases produced by endogenous or exogenous factors.* XRCC1* Arg194Trp, Arg280His, and Arg399Gln single-nucleotide polymorphisms have been shown to have functional significance and could alter* XRCC1* function, decrease the kinetics of repair mechanism, and influence susceptibility to cancer [9, 10].

Because the* XRCC1 *gene polymorphisms may alter DNA repair capacity, a number of studies have suggested that they might represent a risk factor in hematological malignancies such as leukemia [11–14]. Also, the* XRCC1* polymorphisms have been extensively studied in relation to acute myeloid leukemia (AML) [3, 13, 15, 16], acute lymphoblastic leukemia [17–19], chronic lymphocytic leukemia [20, 21], and lymphoma [22–26].

The role of* XRCC1* gene polymorphisms in CML was investigated in only two studies [14, 27]. One study failed to demonstrate an association between* XRCC1* Arg399Gln polymorphism and CML [27]. In contrast, the other study found a significant association of* XRCC1* codons 194 and 399 with CML. However, this was not the case for codon 280 [14].

The* XRCC3* gene product plays an important role in homologous recombination repair of DNA double strand breaks.* XRCC3* Thr241Met gene polymorphism could be associated with impaired function of repair, because this polymorphism consisting of Met to Thr substitution might influence the enzyme's function by removing a phosphorylation site [28].

The* XRCC3* gene has been studied in association with leukemia. Yan et al. found a significant association between* XRCC3* Thr241Met polymorphism and leukemia, in Asian patients [29]. Qin et al. [30] reported that* XRCC3* Thr241Met polymorphism might be associated with AML risk. Seedhouse et al. reported no effect for the variant* XRCC3* 241Met gene alone in either* de novo* AML or therapy-related AML (t-AML) but demonstrated an increased risk of AML when both variants* RAD51* 135C and* XRCC3* 241Met alleles were present [31].

The* XPD* gene (xeroderma pigmentosum group D) is involved in the nucleotide excision repair (NER) pathway. The* XPD* gene encodes a DNA helicase, essential for transcription initiation, nucleotide excision repair, cell cycle control, and apoptosis. Mutations in* XPD* gene reduce helicase activity and cause defects in NER pathway [32, 33]. Single-nucleotide polymorphisms (SNPs) of* XPD* gene, such as Arg156Arg, Asp312Asn, and Lys751Gln, have been studied in relation to lung cancer [32] and colorectal cancer [28, 34]. In the last years* XPD* Lys751Gln polymorphism has been investigated in different hematological malignancies, such as acute myeloid and lymphoblastic leukemia, but with contradictory results [35–41].

There is evidence that variant homozygous genotypes of* XPD* Lys751Gln polymorphism are associated with low DNA repair capacity for benzo(a)pyrene adducts and UV DNA damage [42]. To our knowledge, no data are available regarding the role and distribution of the* XRCC3* Thr241Met and* XPD* Lys751Gln gene polymorphisms in CML.

We focused in particular on the* XRCC1* Arg399Gln, Arg280His, and Arg194Trp,* XRCC3 *Thr241Met, and* XPD* Lys751Gln polymorphisms because they were the most studied and have been shown to be responsible for a suboptimal DNA repair capacity. Thus, they might influence susceptibility to cancer.

The aim of our study was to evaluate the association between* XRCC1* Arg399Gln, Arg280His, and Arg194Trp polymorphisms and the risk of developing CML in Romanian patients. In addition we assessed whether there was an association between* XRCC3* Thr241Met and* XPD* Lys751Gln polymorphisms and CML, as such data are lacking from the literature.

## 2. Materials and Methods

### 2.1. Patients and Controls

The study was performed with approval from the Ethics Committee of the University of Medicine and Pharmacy Tirgu Mures, Romania. The study was carried out according to the guidelines of the Declaration of Helsinki and informed consent was obtained from each participant.

A total of 156 previously untreated adult patients aged 20 to 78 years diagnosed with CML (69 females and 87 males; mean age 51.5 ± 1.1 years) and 180 control individuals (90 females and 90 males; mean age 49.8 ± 2.1 years) were included in this study. The CML patients were consecutively hospitalized and diagnosed in the Hematology Clinics from Tirgu Mures and Cluj-Napoca between December 2010 and December 2013, according to current WHO standards [43, 44]. Controls were randomly unrelated healthy individuals from the same geographical area like the patients (north-western and central parts of Romania), with no previous or present history of malignancy. All patients included in the study had Philadelphia chromosome and/or the BCR-ABL positive CML. Blood samples were collected at diagnosis, before starting therapy. The exclusion criteria were any other cancer types (including other hematological malignancies).

The median hemoglobin (Hb) level at diagnosis was 9.86 g/dL (range, 4–14.1). The median blasts percentage in peripheral blood at presentation was 9.48%. Additional cytogenetic abnormalities (ACA) were observed in 20 CML cases (12.8%). There were 134 patients (85.9%) in chronic phase, 8 patients (5.1%) in accelerated phase, and 14 patients (9.0%) in blast phase.

Patients received first-line therapy with imatinib mesylate (Gleevec), 400 mg/day in chronic phase. In the case of suboptimal response and failure to imatinib treatment the dose was increased to 600 mg/day imatinib, or they received dasatinib (Sprycel) or nilotinib (Tasigna).

### 2.2. Genotyping Procedures

Genomic DNA was obtained from peripheral blood samples using the commercially available Quick-gDNA MiniPrep kit (Zymo Research, USA) and Wizard Genomic DNA Purification Kit (Promega, Madison, WI, USA). The* XRCC1* genotypes were determined by the polymerase chain reaction-restriction fragment length polymorphism (PCR-RFLP) assay. The primers, restriction enzymes, and PCR conditions for* XRCC1* were the same as described by Batar et al. [9], Seedhouse et al. [41], and Wang et al. [45].* XRCC3* Thr241Met genotypes were detected using a PCR-RFLP method, as described previously [46]. The* XPD* Lys751Gln polymorphism was also investigated by a PCR-RFLP assay, as described by Seedhouse et al. [41].

### 2.3. Statistical Analysis

All data were analyzed by GraphPad InStat software, version 3 (GraphPad, San Diego, CA, USA). Fischer's exact test (two-sided) was used to compare the distribution of qualitative variables between cases and controls. A *P* value less than 0.05 was considered as statistically significant. The odds ratio (OR) and 95% confidence intervals (CIs) were used to estimate the strength of the association between alleles and genotype in CML patients and controls. Moreover, the Hardy-Weinberg equilibrium was evaluated using chi-squared test.

## 3. Results

The observed genotype frequencies in controls were consistent with the Hardy-Weinberg equilibrium.

The genotype distribution and the allele frequency of the five polymorphisms analyzed are shown in Table 1. The clinical characteristics of CML patients according to* XRCC1*,* XPD*, and* XRCC3* gene polymorphisms are summarized in Table 2.

We did not observe an association between CML and* XRCC1 *and* XRCC3 *variants. In the case of the three* XRCC1* polymorphisms analyzed, the distribution of the variant heterozygous and homozygous genotypes was similar in patients and controls.

Similarly, in the case of the* XRCC3* Thr241Met polymorphism, the heterozygous and variant homozygous genotypes shared similar frequencies in CML patients and controls. Also, the variant allele frequencies were similar in patients and controls in the case of all* XRCC1* and* XRCC3* polymorphisms analyzed.

In this study, an association between* XPD* Lys751Gln polymorphism and CML was noted. A statistically significant difference was observed in the variant genotype frequencies of the* XPD *Lys751Gln polymorphism between the patients with CML and control group (for variant homozygous genotypes, OR = 2.37; 95% CI = 1.20–4.67; *P* value = 0.016 and for combined heterozygous and variant homozygous genotypes, OR = 1.72; 95% CI = 1.10–2.69; *P* value = 0.019). This was also observed when analyzing the variant 751Gln allele (OR = 1.54; 95% CI = 1.13–2.11; *P* value = 0.008).

We analysed the distribution of* XRCC1*,* XRCC3*, and* XPD* variants in patients stratified by gender. The* XRCC1* Arg194Trp,* XRCC1* Arg280His,* XRCC1* Arg399Gln,* XRCC3* Thr241Met, and* XPD* Lys751Gln polymorphisms studied had no influence on the risk of CML with respect to gender.

We evaluated the impact of these polymorphisms in more detail taking into account different prognostic factors. In the current study, no association was observed in the distribution of any of the* XRCC1*,* XRCC3*, and* XPD* polymorphisms regarding blasts and white blood cells count (*P* value > 0.05 for all these comparisons). When the Sokal and Hasford risk groups were considered, no association was seen between variant genotypes for the* XRCC1*,* XRCC3*, and* XPD* polymorphisms and the risk groups mentioned above.

Finally, we performed a comparison of the ACA with respect to the studied polymorphisms. No association was seen in the distribution of the* XRCC1* Arg194Trp,* XRCC1 *Arg280His,* XRCC1* Arg399Gln,* XRCC3* Thr241Met, and* XPD* Lys751Gln polymorphisms regarding ACA (*P* value > 0.05 for all these comparisons).

Patients with specific genotypes were not more likely to receive a particular tyrosine kinase inhibitor (imatinib, dasatinib, or nilotinib), for none of the polymorphisms analyzed (*P* value > 0.05 for all these comparisons).

## 4. Discussion

In the present research, we investigated the association between* XRCC1*,* XRCC3*, and* XPD* gene polymorphisms and CML in a 6.1 million population from north-western and central regions of Romania. According to the Romanian Association of Rare Cancers the estimated incidence of CML in our country is about 1.6 new cases per 100,000 adults every year [47].

Data regarding the relationship between* XRCC1* polymorphisms and CML are limited, and the results are contradictory so far. Deligezer et al. [27] did not find an association between* XRCC1 *codon 399Gln polymorphism and CML. Annamaneni et al. suggested recently that* XRCC1* gene might have an important role in CML progression but not in its etiology [14].

Our study provides no evidence of a role of* XRCC1* Arg194Trp and Arg399Gln polymorphisms in susceptibility to CML. We found no significant association between the* XRCC1 *194Trp and 399Gln alleles and CML risk. Our findings are not in agreement with the results reported by Annamaneni et al. [14] but consistent with those reported by Deligezer et al. [27].

El-Din et al. [13] observed that subjects with both polymorphisms (*XRCC1* Arg194Trp and* XRCC1* Arg399Gln) have a higher risk of developing AML. Similar results were reported by Joseph et al. [17] in patients with acute lymphoblastic leukemia.

Takanami et al. [48] reported results suggesting that the* XRCC1 *variant 280His allele is associated with a reduced capacity of single-strand breaks (SSB) and BER systems, which consequently increases the risk of carcinogenesis. However, our study did not reveal a statistical significant difference between CML patients and controls, regarding the distribution of the* XRCC1 *Arg280His polymorphism.

Our results are similar to that observed by Zhang et al. in a recent meta-analysis of 19 case-control studies which evaluated the association between* XRCC1* Arg399Gln, Arg194Trp, and Arg280His polymorphisms and leukemia risk. The findings of the meta-analysis demonstrate that* XRCC1* Arg399Gln, Arg194Trp, and Arg280His polymorphisms are not associated with overall leukemia risk, but they could be associated with the risk for some specific leukemia entities [49].

The contradictory results from different studies performed on* XRCC1 *polymorphisms may be due to the ethnic origin, sample size of the studied populations, and different study designs. Also, variation in carcinogenic exposure, alcohol consumption, and cigarette smoking may contribute to differing results.

The frequencies of* XRCC1* 194Trp, 399Gln, and 280His alleles in our CML patients were 0.13, 0.32, and 0.26, whereas in controls they were 0.16, 0.30, and 0.21, respectively. Deligezer et al. [27] analyzed the* XRCC1* Arg399Gln polymorphism on a cohort which included 182 cases of CML and 226 controls from Turkey. The frequency of the variant Gln allele was 0.35 in controls and 0.34 in CML cases. In a recent study Annamaneni et al. [14] explored possible association of the* XRCC1* repair gene (codons 399, 280, and 194 polymorphisms) with CML in 350 patients from Hyderabad, India (South Asia). In the study mentioned above, the frequency of* XRCC1 *Gln, His, and Trp alleles was 0.50, 0.006, and 0.85 in CML patients, whereas it was 0.49, 0.018, and 0.81, respectively, in controls [14]. Thus, the frequencies for the* XRCC1 *399Gln allele and its distribution in the control group were similar to those found in the population from Turkey [27] and less than those in the population from India [14], suggesting ethnical variance. The frequency of the* XRCC1 *194Trp allele was higher, while the frequency of the 280His allele was similar in controls from India [14], compared to those observed in our controls.

We supposed that* XRCC1* polymorphisms do not only increase the susceptibility to CML but also may predispose to developing ACA in CML. When comparing patients with to those without ACA, genotype frequencies of the investigated polymorphisms were not found to be significantly different.

In the current study, no association was seen in the distribution of the* XRCC1* polymorphisms regarding age, gender, and Sokal and Hasford risk groups when comparing wild-type genotypes with variant genotypes. However, we observed an increased frequency of the* XRCC1* Arg194Trp polymorphism among CML patients in accelerated and blast phase. This observation attained a borderline statistical significance (*P* = 0.05).

We also studied the genotype distribution of the* XRCC3* Thr241Met polymorphism in our patients with CML and controls. Our results suggest that the* XRCC3 *Thr241Met variant genotype is not a risk factor for the development of CML. No association was observed between the prognostic factors (age, gender, blast and WBC count, ACA, and Sokal and Hasford risk groups) and the* XRCC3* Thr241Met variant genotypes in patients with CML.

Similar results were reported by Yan et al. in a meta-analysis which included seven studies with 1070 cases and 1850 controls [29]. Yan et al. found no association between* XRCC3* Thr241Met polymorphism and leukemia risk in overall populations, but significant association between* XRCC3* Thr241Met polymorphism and leukemia risk was found in Asians [29].

No significant association was found between the* XRCC3* Thr241 Met polymorphism and the risk of ovarian cancer [50].

These findings are not in agreement with the studies conducted by Voso et al. [51] and Hamdy et al. [52] in which they suggested that* XRCC3* genes polymorphisms might play an important role in the development of AML.

In our study, the frequency of* XRCC3* 241Met allele was 0.36 in CML patients and 0.30 in controls. According to Seedhouse et al. [31], which included 216 cases of* de novo *AML and 186 controls, the variant allele frequencies were 0.29 in controls and 0.34 in AML patients. In the study of Voso et al. [51] the frequency of the variant* XRCC3* 241Met allele was 0.45 in AML patients from Italy. Thus, the frequencies for the* XRCC3 *241Met allele were similar to those found in other Caucasian populations [31, 51].

We also evaluated the potential role of* XPD* Lys751Gln polymorphism and CML risk. Our results suggest a positive association between the* XPD* Lys751Gln variant homozygous (Gln/Gln) and combined heterozygous + homozygous variant genotypes (Lys/Gln + Gln/Gln) and the risk of CML. We also observed an association between variant* XPD* 751Gln allele and the risk of CML. These results suggest that the* XPD* Lys751Gln polymorphism may contribute to leukemogenesis in CML. Our findings are in agreement with the study conducted by Özcan et al. [36]. They suggested that variant* XPD* 751Gln allele is associated with a reduced DNA repair capacity and increased leukemogenic risk and that* XPD *Lys751Gln polymorphism may affect the outcome of childhood AML therapy [36]. Similar results were also reported in previous studies, in which* XPD* Lys751Gln variant genotypes were shown to be risk factors for AML [37] and acute lymphoid leukemia [38]. These findings are not in agreement with the study conducted by Sorour et al., in which they found no differences in the frequency of the* XPD* Lys751Gln polymorphism between AML patients and controls [35].

The frequency of* XPD* 751Gln allele was 0.42 in our CML patients and 0.32 in controls. According to Wang et al., the frequency of the* XPD* 751Gln allele was 0.39 in Europeans, 0.36 in Americans, 0.12 in Asians, and 0.24 in Afro-Americans [38]. In a meta-analysis performed on 56 case-control studies, the Gln/Gln variant genotype of the* XPD* codon 751 was associated with increased cancer risk compared with the Lys/Lys genotype only in the European population [38]. The variant* XPD* 751Gln allele frequency was 0.38 among the controls, while it was 0.30 in AML patients from Egypt [35].

In conclusion, our study suggests that the* XPD* Lys751Gln polymorphism increases the risk of CML. According to our findings, the* XRCC1 *Arg194Trp, Arg280His, Arg399Gln, and* XRCC3* Thr241Met polymorphisms are not a risk factor for CML.

In the future, similar studies performed on larger cohorts of patients should clarify the relationship between* XRCC1*,* XRCC3*, and* XPD* polymorphisms and CML.

## Figures and Tables

**Table 1:** Genotype distribution and allele frequency for the five polymorphisms analyzed in patients with CML and controls.

Polymorphism		CML patients *n* (%)	Controls *n* (%)	OR (95% CI) CML versus controls	*P* value CML versus controls
*XRCC1* Arg194Trp	Arg/Arg	119 (76.3)	129 (71.7)	—	—
	Arg/Trp	31 (19.9)	45 (25.0)	0.75 (0.44–1.26)	0.294
	Trp/Trp	6 (3.8)	6 (3.3)	1.08 (0.34–3.45)	1.00
	Arg/Trp + Trp/Trp	37 (23.7)	51 (28.3)	0.78 (0.48–1.28)	0.384
	Arg allele	269 (86.2)	303 (84.2)	—	—
	Trp allele	43 (13.8)	57 (15.8)	0.85 (0.55–1.31)	0.515

*XRCC1* Arg280His	Arg/Arg	82 (52.7)	112 (62.2)	—	—
	Arg/His	64 (41.0)	58 (32.2)	1.51 (0.96–2.38)	0.083
	His/His	10 (6.3)	10 (5.6)	1.37 (0.54–3.43)	0.636
	Arg/His + His/His	74 (47.4)	68 (37.8)	1.48 (0.96–2.29)	0.077
	Arg allele	228 (73.1)	282 (78.3)	—	—
	His allele	84 (26.9)	78 (21.7)	1.33 (0.93–1.89)	0.124

*XRCC1* Arg399Gln	Arg/Arg	71 (45.5)	91 (50.6)	—	—
	Arg/Gln	69 (44.2)	73 (40.5)	1.21 (0.77–1.91)	0.421
	Gln/Gln	16 (10.3)	16 (8.9)	1.28 (0.59–2.74)	0.563
	Arg/Gln + Gln/Gln	85 (54.5)	89 (49.4)	1.22 (0.79–1.88)	0.382
	Arg allele	211 (67.6)	255 (69.7)	—	—
	Gln allele	101 (32.4)	105 (30.3)	1.16 (0.84–1.62)	0.401

*XPD* Lys751Gln	Lys/Lys	51 (32.7)	82 (45.6)	—	—
	Lys/Gln	77 (49.4)	79 (43.9)	1.57 (0.98–2.51)	0.075
	Gln/Gln	28 (17.9)	19 (10.5)	2.37 (1.20–4.67)	**0.016**
	Lys/Gln + Gln/Gln	105 (67.3)	98 (54.4)	1.72 (1.10–2.69)	**0.019**
	Lys allele	179 (57.4)	243 (67.5)	—	—
	Gln allele	133 (42.6)	117 (32.5)	1.543 (1.13–2.11)	**0.008**

*XRCC3* Thr241Met	Thr/Thr	64 (41.0)	85 (47.2)	—	—
	Thr/Met	70 (44.9)	79 (43.9)	1.17 (0.74–1.86)	0.561
	Met/Met	22 (14.1)	16 (8.9)	1.82 (0.88–3.76)	0.105
	Thr/Met + Met/Met	92 (58.9)	95 (52.8)	1.28 (0.83–1.98)	0.272
	Thr allele	198 (63.5)	249 (69.2)	—	—
	Met allele	114 (36.5)	111 (30.8)	1.29 (0.94–1.78)	0.120

**Table 2:** Patient features at diagnosis according to the *XRCC1, XPD*, and* XRCC3* genotypes.

	Overall	*XRCC1* Arg194Trp			*XRCC1* Arg280His			*XRCC1* Arg399Gln			*XRCC3* Thr241Met			*XPD* Lys751Gln		
		Arg/Arg	Variant*	*P*	Arg/Arg	Variant*	*P*	Arg/Arg	Variant*	*P*	Thr/Thr	Variant*	*P*	Lys/Lys	Variant*	*P*
Gender																
Female	69 (44.2)	51	17	0.849	42	27	0.076	33	36	0.630	30	39	0.624	26	43	0.302
Male	87 (55.2)	68	20		40	47		38	49		34	53		25	62	
Age																
<50 years	68 (43.6)	55	13	0.259	34	34	0.629	37	31	0.053	32	36	0.192	22	46	1.00
>50 years	88 (56.4)	64	24		48	40		34	54		32	56		29	59	
Clinical phases																
CP	134 (85.9)	106	28	**0.056**	72	62	0.498	66	59	0.104	59	75	0.065	40	94	0.084
AP/BP	22 (14.1)	13	9		10	12		7	15		5	17		11	11	
Sokal risk groups																
Low	64 (41.0)	44	20	0.085	28	36	0.746	30	34	0.870	27	37	0.870	21	43	0.098
Intermediate	52 (33.3)	45	7		27	25		30	22		20	32		24	28	
High	40 (25.7)	30	10		16	24		11	29		17	23		19	21	
Hasford risk groups																
Low	55 (35.2)	42	13	1.00	25	30	0.240	22	33	0.318	25	30	0.496	19	36	0.724
Intermediate	65 (41.7)	53	12		35	30		33	32		21	44		22	43	
High	36 (23.1)	24	12		22	14		16	20		18	18		10	26	

CP: chronic phase, AP: accelerated phase, BP: blast phase, and variant*: heterozygous and homozygous variant genotypes.
